# Metallic Posts Enlarged to Fit Badly Decayed Root Canals

**Published:** 1908-09-15

**Authors:** B. J. Cigrand

**Affiliations:** Chicago, Ills.


### METALLIC POSTS ENLARGED TO FIT BADLY DECAYED ROOT CANALS.

BY B. J. CIGRAND, M. D., D.D.S., CHICAGO, ILLS.

Read before the Southwestern Michigan and Fifth District Dental Societies, April 14 and 15, 1908.

The dental profession during the past five years has devoted much attention to crown and bridge work, the consideration of the superstructure, root preparation, band construction and crown formation. We have arrived at a point where we fully realize the importance of the mechanical principles involved, in that we appreciate the force of the jaw and the necessity of perfect fit on the roots. In this consideration of force, we have, in a measure, forgotten the importance of the basic purpose which determines the ultimate success of the mechanism. Imperative as it may seem to fully comprehend the superstructure, the substructure of the crown or bridge, its anchorage is indeed primal. The one is no more essential than the other and both are indispensable.

In the past we have been concerned in the construction of the bridge and have practically lost sight of its fundamentals, and if its pillars of location are disregarded, discouraging failure must be the climax.

In my mind the opinion exists that there is still a disposition on the part of too many operators to employ the forceps whenever the dental organs present conditions slightly unfavorable for a filling, inlay or crown. There is much comfort, however, in the fact that a happier era is upon us, for the dentists are in eager search for a method or system which will make it possible to restore badly decayed roots and restore the dental arch. The general public is gradually educating us to a full realization of the importance of saving teeth, and the time is not far distant when "the loss of a good tooth will bring sorrow to the entire household."

It is deplorable that too frequently the lateral or first bicuspid roots are extracted, and a tooth supplied by some system of bridge work, cutting down the sound adjoining teeth, collaring them with gold, when it was within the province of dental prothesis to save the root, and place upon it an individual crown, such as would afford the natural denture with a hygienic substitute fully in accord with physiological laws. Nature scorns to be tied up or confined, and the present system of bridge work, with their yokes of gold, are contrary to nature and work ill results—more especially when the load is unbearably large, as in the case of an attachment of five or eight artificial teeth resting on two roots. How pre-eminently better to save these roots by affixing individual crowns. To avoid applying bridge work in every instance possible and attach the individual crown instead, should be the earnest ambition of every member of our profession, yet some continue advocating large bridges on weak foundations and inefficient anchorage.

The individual tooth as nature supplies it is independent of its neighbor, being only in touch at what is known as the contact point, and the principle of mastication can be shown as complete in one superior and two inferior teeth, or vice versa. That we should all strive to preserve this separateness must appeal to all who value nature as a model in creation.

We are all applying bridges where individual crown work would be far more logical. Bridge work has suffered because of its injudicious employment, but that is the usual complaint against all new discoveries or inventions. Eagerness to employ the find, or creation, has lead to the discontinuance of many a praiseworthy appliance or medica-cation. Enthusiasm is a necessary attribute in all progressive work, but it must be curbed by a judgment founded on experience. That bridge work has suffered much through too prevalent applications, no one familiar with its history will dispute. Advances in the arts and sciences must be of slow growth. Like government affairs, reform must be of so gradual process that, unbeknown, it steals its way into the hearts of men; for when of too rapid a nature it is both unhealthy and shortlived. In our profession, as in all organized society movements, the conservative must be the "pillars of strength."

The conservatiye practitioners throughout the land have recognized the error of the enthusiastic bridge builder. They have observed that the hastily constructed Richmond crown with ill-fitted band has inaugurated a siege of peri-dental and alveolar troubles. They have noted that the all-gold-telescope advocate, who caps a slightly decayed tooth, has outraged his profession.

Dental annihilation properly belongs to the oral surgeon and has nothing in common with prosthetic dentistry, which has come to mean dental substitution and preservation. Crown work has advanced in theory only when the theory has brought fruit in actual practice. In its two-fold evolution it absorbs from every available source which tends to broaden its art or perfect its science, and in consequence calls to its aid mechanics and medicine.

In a prominent dental society not long since, the question of saving bad roots was discussed, and some few entertained the idea of using gutta-percha, advocating that this material admitted of easy removal of the crown and also acted as a soft, yielding cushion for the porcelain substitute. I am not prepared to accept this method as practical or hygienic. I doubt the practicability of such a procedure, besides it indicates a temporizing effort. But this is not the main reason for my objection to the use of gutta-percha alone; I cannot believe that a crown which is set on a material which acts as a cushion is sufficiently dense to exclude the oral fluids. If it yields, springs and changes to every form of pressure, it goes without saying that the material is porous and in consequence harbors the oral fluids, and this would quickly destroy its efficiency, and also allow lodgment of debris, I have tried this method and have discarded it since I have greater faith in the employment of cement in combination with metal. If any practitioner depends on gutta-percha may I ask that the next case of crown or bridge work he is called upon to reset he will do himself a great favor by subjecting the removed piece with its aged gutta-percha to a microscope; that ocular object lesson will suffice as to the cleanliness or efficiency of this deteriorating, temporizing material.

If I have convinced you that these badly decayed roots can be saved and made normally useful, I shall feel that my years of thought and study on this line have not been in vain. The method I have incorporated in several papers and clinics during the past ten years, though not until within the past two years have prosthetic operators appreciated the principle underlying my suggestion.

If the root or roots in question are still possessed of sound or healthy tooth substance there is no reason why they should be extracted; if you can attach to them a firmly anchored, well-fitting crown and accord to that root the natural exercise of mastication, you restore to its peridental membrane a renewed circulation of blood and in turn recreate a vigorous dental antagonism, the basic principle of sound dental organs. While, if the root is allowed to remain uncrowned and its exposed portion is denied contact, certainly nature will inaugurate an effort to throw off the useless member; but, observe the rules of mechanics, and apply a crown to this apparently drone root—the change which proceeds is astonishing and the prosthesis is perfect.

The method is simple and effective. Prepare the badly decayed root as one in ordinary practice, ream out all the broken-down tissue and depend on sound structure, fit the crown, be it Logan, Davis, Brewster or Justi, in the usual manner; just before setting same apply a bit of wax to the metallic post and insert in the cosmolined canal. At first introduction you may have an excess of wax; remove, and over alcohol flame soften and trim where indicated, then reinsert and after getting a good, accurate adaptation, the initial step is complete. Then take the crown with its wax-encased post and invest entirely in the ring of your casting apparatus and after freeing the wax from investment and being assured of the perfect dryness of the case proceed to liquidate the gold and by means of air, steam, or better still, suction, cast the case. While gold is the metal indicated I must confess that after diligent investigation, Acolite will practically give the same results, in that as a pure metal it is acceptable to the tissues and gives us all the desirable qualities so essential in a metal permanently located in the mouth.

There are innumerable cases where the mesial or distal surfaces of roots are broken down, and by this method all you need to do is to drill away the decayed portion, adapt the wax and cast as already indicated.

In shaping root canals for crowns or bridges it is a mistake to employ a large drill and produce a large circular opening into the root to receive the metal post. Choose a small reamer, and by giving it an anterio-posterior movement you are enabled to cut an opening of an elliptical character, and you leave the root structure thick at its lateral sides, where the major strain falls and where the root must of necessity be the strongest. Further, this rhomboidal opening thus affords additional anchorage to the crown.

It is evident that a crown set as recommended cannot loosen or fracture the root unless the post first stretches, and this, I believe, is the cause of many of our crowns loosening. The primary cause does not lie hidden in this, however, but in a factor, of which I will speak later. If the posts in the Logan, or in any of the full porcelain crowns, were made of iridio-platinum instead of pure platinum there would be less likelihood of the yielding process and the stability of the crown would be more assured.

This form of casting so accurately fits the canal and adjacent parts that independent of cement the crown would be held firmly in position, but a film of cement, or insoluble varnish or fluid, the crown is definitely lodged.

Then there are cases not infrequently met where the anterior half of the root is partially broken away and these cases tax the ingenuity of all operators, but by the method I advocate, these distressing cases are made simple indeed, Just pack cotton or gutta-percha into the space crowding away the gum tissue, then dismiss the patient for an appointment on the following day, when full and easy access is accorded to the space, and after packing the wax both in root canal and broken anterior aspect, you remove the entire mass of wax and cast as already noted.

In the event you wish to employ facings to harmonize with remaining teeth, the method is also very adaptable, since the facing is ground to fit, being backed up by wax, and after removing entirely you have produced a very acceptable crown and not only saved a bad root but restored a denture.

It may seem like an extreme statement, but this method should be employed in the setting of every crown and bridge, since it gives promise of permanent work. Besides it is an accord with the science of mechanics, an element which deserves our constant attention.

The all-gold shell, when telescoped over a large amalgam filling often results in failure because of the action of the mercury in the filling, and when this filling is anchored by screws other than gold or platinum, it will invariably result in failure, notwithstanding that the gold crown was constructed without a fault. The anchorage was insufficient.

In a case of this character just adapt the wax and build same to conform to a natural crown properly shaped, then remove and cast, set same with cement and you have formed an anchorage for the gold crown not only congenial with the surrounding tissues but absolutely compatible with the gold telescope it is to receive, since Acolite in this instance is pre-eminently indicated because of its virtues in conjunction with gold and tissue.

Now this method of restoring badly decayed roots is not advocated by me because of its productions of restorations in a simple way, though it does accomplish this event but because it conserves dental organs and brings to our enfeebled humanity an element of redress and accords to the philosophy of mastication--an attribute indispensable and yields pronounced results.

DISCUSSION.

DR. A. C. RUNYAN: No doubt all of us have tried at different times in our lives to bring about results as suggested by Dr. Cigrand. In my practice I have tried a great many times to bring about this result with amalgam, by putting in posts and building up the amalgam, but I have never been fortunate enough to get good smooth results. They always left places for debris to collect and for irrititation, or produced irritation. Dr. Cigrand puts this operation into this thorough and concise form which it seems to me is very simple. It opens up a new field to us. The nearest thing that I ever saw to it was at the National Dental Association, in Niagara Falls, several years ago, Dr. Carmichael, of Milwaukee, presented a clinic something along this line. He took the broken-down teeth very much in the same way and used platinum and inserted it into the cavity and enlarged it and built on to that post ,and this was all filled with solder; but in this case with the casting machine, as I understand Doctor Cigrand, all these things are eliminated and you have the whole thing made at one fell swoop, as it were. These beautiful drawings— and, by the way, I think Doctor Cigrand is an adept at drawing— show every step of the method so thoroughly that there is no point left open for discussion. So far as the technique is concerned, it does certainly seem to me that it has brought about one of the most perfect ways of restoring teeth. Now it is astonishing what badly broken-down roots will stand, I have been in practice for nearly

30 years and I have restored or have put Logan crowns on teeth that it seemed would stand only a very short time, and it has been astonishing to me to see how long they will stand being used. I have always been a very great friend of the Logan crown. I believe it one of the greatest inventions in dentistry from the fact that you can restore a single tooth in a way that is certainly artistic and very useful. By the way, I have set the old wooden-pin crowns when we had to depend upon the swell of the wood to hold them. But as soon as the Logan crown made its appearance I adopted them. The great fault that he speaks of regarding the Logan crown is in the incisors that almost invariably have the condition that he speaks about. For that reason I have discarded using the Logan crown for lateral incisors. However, by strengthening them in the manner he speaks of, they can be used. Now the reason why I am so pleased with the Logan crown; those who have porcelain can color them in any way they have a mind to, they can change the shade of them, they can put on the streaks or grind them up and you can get very artistic results, while with some of the other crowns you cannot. Possibly with the Davis crown you can use it in the same way. But I have used the Logan crown so long that I think I can set i t more readily than almost any other crown there is. For lateral incisors I have been using the 20th Century crown for some time. Bicuspids that are broken down are difficult to replace with the porcelain crown; often the walls are broken down so badly that you cannot get a satisfactory result in crowning in any way, and when attempting to remove the roots, or to swing the tooth onto the next bicuspid, we almost always find that in the case of a few years it is sad failure. In this manner you can save the bicuspids and they will be of good service for a great many years.

Dr. N. S. Hoff. There is one feature of this work that I wish to emphazise. Technically, I find no fault with the method employed. And I wish to commend any effort in the direction of saving roots of teeth. My remarks a few moments ago in regard to the paper on orthodontia were to the same point. If I have a hobby it is saving teeth. There are few roots of teeth that are so bad that they cannot be cured and utilized in some way, unless, of course, there is a condition of necrosis about them that makes them a menace to the health of the patient. A great many times we extract roots of teeth that we think are of no special value in order that we may supply bridge work, as Doctor Cigrand has said. And I wish to say in parenthesis here that I am not exactly in harmony with Doctor Cigrand's idea that we should never make bridge work. I don't know as he meant that exactly, but I think very often a root or tooth that has a very slender or precarious attachment to the process can be made substantial and valuable in mastication if it is attached to an adjoining tooth which has a better and a stronger support, and be made much more useful than if it were crowned alone and had to depend upon its own strength and support for its use. I think bridge work fills a very important and essential place in our work and I am not ready now to abandon it; but this plan of saving broken-down, or what are apparently hopelessly decayed roots which don't appear to be of much consequence or value, is a great thing. Now of what use would be this beautiful work to which Doctor Land has called our attention today, if it was to be put upon roots of teeth that were loosened and coming out? The roots of the teeth are really the important elements of the teeth. The crowns are not valuable in comparison with the roots. It is the roots of the teeth that are the foundations upon which we build the masticatory apparatus, and unless we save the roots of the teeth by all or any means, how can we make the beautiful restorations that Doctor Land and Doctor Cigrand have called to our attention? It is a waste of time and skill to do any of the high grade technical work on worthless or badly diseased roots of teeth. We must put these roots in good, healthy condition first, and this is our chief work in dentistry; it seems to me it is fundamental and basic to all other successes. I believe we are losing sight of the value of the roots of the teeth in our efforts to beautify the portions of the teech that show, and are conspicuous in the mouth. I want to commend Doctor Cigrand's paper on that point, if no other. All roots, however badly decayed they may be, unless hopelessly diseased, are capable of restoration to health and to do great service and usefulness by Cigrand's method, or Land's, or by some other such method, and it seems to me this is going back to the fundamental point, and we must save all the roots of the teeth. Save every root and do not abandon any root until you are thoroughly convinced or satisfied that it cannot be made useful, because it would have to be a pretty bad root that could not be put into good form and use by this method that Doctor Cigrand has suggested to us.

Dr. C. H. Land. I am always glad to hear anything from Doctor Cigrand, and also from Doctor Hoff, in his remarks on this subject. The remarks that I would like to make on this work here particularly is the adaptation. It has been my practice for years in all my work to use the minimum amount of cement and have always been particular in that regard. I have taken especial pains and I have shown a good many how to take an impression of the root clear to the end so that they could make good work of it. There is one thing that is going to make Doctor Cigrand's method successful, and that is it is simple and inexpensive and substantial, and an excellent principle also is to use the minimum amount of cement. It is the excessive amount of cement that makes such work unsuccessful. When Doctor Capon, of Philadelphia, came to me to take instruction in porcelain several years ago, I gave him a very prominent and wealthy man in Detroit for his first patient. To teach him the idea of putting the old roots in good condition, I asked him to take the case and treat every one of the several badly decayed roots because his patient had a good constitution and the only reason why the roots were badly decayed was because they were not in use, seven or eight of them. Any one who would look at them would say, "Get them all out." I insisted on him taking great pains to treat them and finally he treated them and put on porcelain. I have never told Doctor Capon the result of that work. That work was in use just exactly 18 years. The man started for a trip around the world and dropped dead of heart disease in a restaurant at San Francisco, but all the teeth were there. It is all because they were put into use. You let any part of the body be out of use and it will begin to decay. You put it in use and it immediately hardens and braces right up. If you can get the roots into use they will be all right.

In regard to bridge work, I agree that the more you can individualize teeth, unless where they have absolutely no support, as Dr. Hoff has said, you are much better off. If I can make a bridge in four sections, I would rather do it. I do not want to claim that we are not doing better bridge work than the ancients did, because we are, but they needed it in their time, and they bound teeth up with wire, showing that they were troubled with pyorrhea. I wish to congratulate Doctor Cigrand on his neat, simple, practical way of making this work. I want to ask him one question, however; I want to know how he gets the cement down deep in those roots and knows that he gets it there before he puts in the post?

Dr. R. C. Brophy. In the first place I want to express my appreciation of the courtesy extended to me in asking me to take the floor. I have met with you several times before; I have always had a good time. I am having a good time here at the present time. I attend a good many meetings necessarily, but I never yet attended a meeting that I have thought there was quite as much strenuousity exhibited on the part of the presiding officer as I have seen in connection with this meeting.

During the last 50 years of the history of dentistry a number of very prominent and important innovations have been introduced into the practice. Even the younger men present will recall the more prominent ones of these innovations. For instance we have had the introduction of the vulcanizers, a great curse to dental art and skill. We have had the introduction of crown and bridge work, or at least the modernizing of crown and bridge work. We have had the innovation and change, the introduction of porcelain work. We have had the introduction of gas and gold inlay work. Various results have followed these different innovations. from the standpoint of the benefits which l ave accrued to the dental profession and to humanity.

In regard to the introduction of crown and bridge work, or the modernizing of it—of course I cannot go back to the time when the bridge was made as the doctor just spoke of, because I was not there at that time—I would say in regard to bridge work that I am very much in accord with some things which Doctor Hoff said; I believe that bridge work has proven a blessing to humanity. I know that it has proven a blessing to the dental profession. Why? Because it has led up to a higher standard of mechanical skill in the dental profession. Now we pass along to porcelain work. I believe, ladies and gentlemen, that porcelain work has proven one of the greatest blessings to both humanity and the dental profession of anything that we have ever had. It has proven a blessing to humanity because it has led people to regard the science of taste, beauty and the philosophy of fine art; it has brought out for the benefit of the profession perhaps greater mechanical skill, not only greater mechanical skill, but greater artistic skill, than anything else that we have ever had introduced. I don't know but I might better not go any further. Cast gold inlay work in my humble opinion, when confined strictly to decay in the crown of the tooth, will prove a blessing to humanity. On the other hand I am very much afraid that it will prove a curse to the profession of dentistry from the standpoint of mechanical and professional skill.

I believe that the idea of restoring badly broken-down unfortunate roots of a tooth, that like the maiden that

Doctor Blackmarr spoke of a while ago, who longs for the embrace of a strong arm, with a cold piece of steel at the end of it; when we can restore those teeth, I believe we shall find this process one of the greatest blessingsto humanity and one of the most important, if not one of the noblest and kindest services that the dentist could possibly render. I believe that in years to come, when you look back at the introduction of this work of restoring broken-down, hopeless cases of decay by this casting process, you will find it to be a most important and valuable achievement. I believe that the simple matter of casting fillings for cavities in the crowns of teeth will dwindle into insignificance when the entire field, including the restoration of broken-down roots, is known. I congratulate this society upon the fact that Doctor Cigrand has made this meeting the office for the introduction of this process. Doctor Cigrand has of late months taken this matter up with fresh vigor and he has developed a system of doing this work that I know when you return to your homes to your respective practices and take it up you will be very grateful to him.

Doctor Cigrand. Your kind commendations come to me rather unexpectedly because I had anticipated some serious criticisms. Seeing that I do not get this, I cannot see that there is anything for me to say other than that I am glad, indeed, and grateful to you for your generosity in having, at least in my opinion, had the intelligence to grasp the principle at stake. The method has been given in clinics before, but after 7 or 8 years of actual practice in my office I know it is one of the most valuable things; and I want to tell you, Mr. President, that the profession is being educated by the patients. Don't think for one moment that we are educating the public altogether. I want to say frankly and freely, and I want to endorse it with all my heart, that my practice and my patients are my kindest teachers. They are the ones that say to me, "Doctor, can't you save that tooth?" They inspire me with an ambition that means something. When a lawyer comes to me and has a broken-down central tooth and I suggest that it ought to be extracted, never before any living jury has his voice quite that meaning when he looks into my face and says, "Doctor, can't you save that tooth?" That is the thing that educates us and these are the things that we are asked to do every day of our practice, to save teeth. That is our business and should be our ambition.

Conservation, preservation, restoration, that is modern dentistry; extraction is all wrong, it is surgery, it doesn't belong to dentistry. I want to say, and I want to emphasize it with all my heart, that our patients are our kindest teachers if we would but listen to them, for a man that does not get the right impulse from his practice is not in love with it. And it is my patients, and it is your patients that make you climb hills and scale mountains and lie sleepless nights thinking what shall I do to save those teeth, preserve their beauty and restore that health—what a nobler mission! The lawyer, the minister and the physician all have their glorious places, but we are today the most exact, the most demonstratable profession that there is; we demonstrate to the public what we do, they don't have to believe it by Christian Science, they get it by the optic nerve, they see it, and they feel it.

Now then, let me say in conclusion, Doctor Hoff is a dear old friend of mine; we have been together, saddled together and been marched out together in various kinds of conventions, national, interstate and everything else, and I always value very much what Doctor Hoff says. And I wish in kindness to Doctor Hoff to correct just one little statement as it goes down in this report. This sentence I wish to go down in the discussion. "To avoid applying bridge work in every instance possible; to attach individual crowns instead, where nature so indicates it, should be the ambition of every practitioner."

Doctor Land has asked how I know that I get the cement up into the root before I shove the metal post down into position. Just before introducing that metallic post, by a little brush made of platinum with about 5 or 6 apparent bristles of platinum, I take that cement, when it is still in the cream of consistency, and paint down all the root canal surfaces before I introduce the post, and then I put a very sparing amount of cement on the metallic post and I quietly force it into position and I am satisfied that the cement is up to the apex because I have introduced the cement before I put the post in.

This paper is the result of years' experimenting, and while this may seem like a curiosity to you, and is known by my students as my "old pipe", it was the beginning of this casting idea, as it contained the original principle. With this grappling pair of pliers, the asbestos, or any other material, was grappled when the metal was in a molten condition and all I had to do was to get air pressure, which I did by shutting this valve, and the case was cast. I want to say that I certainly would not be inconsistent enough to come here and for one minute claim that I was the originator of casting metal under pressure, because that is as ancient as the hills. I saw Doctor Carrol, 20 years ago, cast some cases of aluminum under pressure, and we find in the records of ancient France that a patent was taken out in 1400 where a man cast iron under the pressure of a large piston. This is not a question of who invented casting by pressure, because there is no man living today in the world who can justly claim the invention. The man that invented it has long since gone to dust and "is nourishing some posy somewhere," as Shakespeare says. The idea is, is it of service to humanity? If it is, let us take it home to our heart to serve that for which we stand.

